# A Novel Approach to Estimate the Plastic Anisotropy of Metallic Materials Using Cross-Sectional Indentation Applied to Extruded Magnesium Alloy AZ31B

**DOI:** 10.3390/ma10091065

**Published:** 2017-09-11

**Authors:** Mingzhi Wang, Jianjun Wu, Hongfei Wu, Zengkun Zhang, He Fan

**Affiliations:** School of Mechanical Engineering, Northwestern Polytechnical University, Xi’an 710072, China; wangmz_nwpu@foxmail.com (M.W.); WuHongfei321@163.com (H.W.); 18292068849@163.com (Z.Z.); henwpu@foxmail.com (H.F.)

**Keywords:** indentation, plastic anisotropy, parameter identification, metallic materials

## Abstract

In this paper, a methodology is presented for obtaining the plastic anisotropy of bulk metallic materials using cross-sectional indentation. This method relies on spherical indentation on the free edge of a specimen, and examining the out-of-plane residual deformation contour persisting on the cross-section after unloading. Results obtained from numerical simulation revealed that some important aspects of the out-of-plane residual deformation field are only sensitive to the extent of the material plastic anisotropy, and insensitive to strain hardening, yield strain, elastic anisotropy, and the selected displacement threshold value. An explicit equation is presented to correlate the plastic anisotropy with the characteristic parameter of the bottom shape of residual deformation contour, and it is used to uniquely determine the material plastic anisotropy in cross-sectional indentation. Effectiveness of the proposed method is verified by application on magnesium alloy AZ31B, and the plastic anisotropy parameter obtained from indentation and uniaxial tests show good agreement.

## 1. Introduction

Indentation testing has long been used as a simple and effective method to extract the mechanical properties of materials, e.g., hardness [1,2,3]. Recent advances in depth-sensing instrumented indentation equipment and optical profiling techniques have greatly stimulated the tremendous interests in characterization of various mechanical properties of materials, e.g., elastic modulus [4], stress strain curves [5,6], fracture toughness [7,8], wear properties [9,10,11], as well as the material anisotropy [12,13,14,15,16,17].

In the nature and synthetic material systems, the anisotropic materials are often observed and widely used in industrial products, such as rolled sheets, composites, extrusions, and so on [18]. The plastic anisotropy is a very important factor that should be considered in the material design and application stages [19,20,21]. On the one hand, the plastic anisotropy has non-negligible influence on the final shapes of metal products made by pressure forming, e.g., the in-plane plastic anisotropy is tightly related to the tendency of rolled sheets to form ears during drawing [18,22]. As one example, magnesium alloys have received significant attention in industrial applications because of the excellent physical properties, e.g., light weight/high strength [23,24]. However, it was reported that this material has relatively poor ductility and obvious plastic anisotropy at room temperature because of the pronounced basal-type texture, e.g., magnesium alloy AZ31 [24,25]. Therefore, a large number of prior works were dedicated to the improvement of the formability and performance of magnesium alloys, such as the usage of different rolling techniques [26,27,28], and the introduction of rare earth [29,30].

On the other hand, the anisotropy of static mechanical properties is tightly related to the micro-texture and micro-structure of materials [31,32,33,34]. For the lithium aluminum alloys extrusion, the deformation texture created during shaping usually causes high levels of anisotropy, such as the asymmetric flow and in-plane plastic anisotropy [32,33,34]. In aerospace applications, a lower strength in the off-axis direction will neglect the benefits of high strength in the working direction, e.g., the wing skins and fuselage stringers [32,33]. Therefore, it is especially important to pay attention to the mechanical characterization of material plastic anisotropy.

The traditional method for the measurement of material plastic anisotropy relies on the uniaxial experiments along different orthogonal directions, e.g., uniaxial tensile/compression tests. However, this testing method is not applicable for the specimens with finite volumes [16]. Therefore, researchers are seeking alternative ways to extract the material plastic anisotropy, e.g., using indentation, by examining the non-uniform surface displacement distribution around indenter [16,17,35]. Additionally, many researchers [13,14,15] resorted to the sophisticated numerical optimization algorithms to calibrate the prescribed anisotropic model, e.g., Hill’s criterion [36], by minimizing a suitably-defined error norm between experiment and simulation. However, the inverse parameters identification processes in these methods usually involve complicated parameters regression and the extensive off-line numerical simulations [16]. It is noted that the stress field induced by indentation is essentially under multi-axial state. Therefore, the indentation actually reflects an “averaged” material response [12,37]. For the anisotropic materials, the indentation response is determined by all the constitutive parameters of the postulated constitutive law. Thus, it is challengeable to correlate solely the plastic anisotropy parameter with a specific material response amount, without taking account of the influence of the other constitutive parameters.

In this paper, we proposed a novel method to estimate the plastic anisotropy parameter of metal materials by examining the out-of-plane residual deformation field in the cross-sectional indentation. The advantage of this method is that the shape of the out-of-plane deformation contour induced by cross-sectional indentation is only sensitive to the material plastic anisotropy, and insensitive to strain hardening, yield strain, and elastic anisotropy. Therefore, the extent of material plastic anisotropy can be uniquely correlated with the indentation response, e.g., out-of-plane residual deformation field.

## 2. Experiment Investigation

### 2.1. Material

The material studied here is the magnesium alloy extrusion AZ31B. This material is widely used in industrial applications for its light weight/low density, and good mechanical properties [23,24]. The chemical composition of this alloy is listed in Table 1, and it refers to the mass percentage. The original shape of the material sample is rounded bar, and the extrusion direction is along the axis. Due to the extrusion process, this material exhibits strong plastic anisotropy. Uniaxial compression experiments were used to investigate the plastic anisotropy of this alloy so that the plastic anisotropy parameter estimated from indentation and uniaxial tests can be comparable.

The compression experiment was implemented on the universal material test machine (CIMACH, Changchun, China), respectively, along the longitudinal and transverse directions. Specimens used in the uniaxial tests were cylindrical, with diameters of 10 mm and heights of 12 mm. Strain during the compression process was measured with the assistance of strain gages and data acquisition devices. The maximum compression ratio was about 10%. Stress–strain curves in uniaxial compression processes were fitted using the Hollomon hardening law. The uniaxial experimental result is listed in Table 2. In this table, the symbols “1, T” and “3, T” represent the transverse direction, while “2, L” represents the longitudinal direction. Results show the yield stress of this material exhibits strong anisotropy along the longitudinal and transverse directions, and the yield stress ratio between longitudinal and transverse directions is about 1.51, while the elastic modulus and strain hardening exponent show very slight anisotropy.

### 2.2. Cross-Sectional Indentation

**Figure 1:**
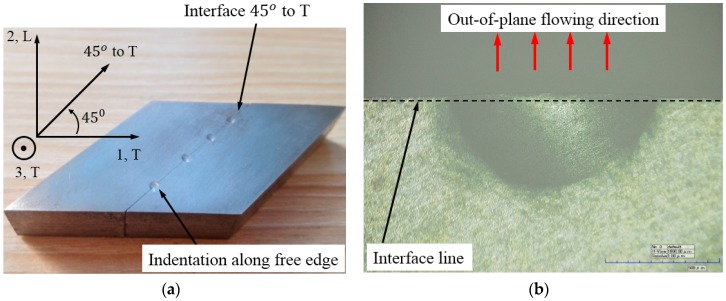
(**a**) Specimen used in cross-sectional indentation experiment; and (**b**) the residual imprint left on the surface of specimen.

Figure 1a shows the material coordinate and specimen used in cross-sectional indentation experiment. The specimen was cut along the diagonal direction (45° to the transverse direction) in the T-L plane. Then the two cross-sectional surfaces were carefully polished to a mirror finish and glued together. The rapid glue used in the indentation experiment was a soft type, and the glue thickness was less than 20 µm. The top and bottom surfaces of the specimen were flat, in order to avoid the tilt of the specimen in the indentation loading process. Spherical indentation was implemented on the hardness tester at room temperature, and the indenter was a tungsten ball with a diameter of 5 mm. The indenter was vertically pressed against the free edge along the bounded interface, so that the material under the indenter is able to flow freely along the out-of-plane (vertical to the cross-sectional surface) direction. Before indentation, the surface of the specimen was carefully polished to a mirror finish. Two different maximum indentation loads, Load-1: $P_{max}$ = 400 N and Load-2: $P_{max}$ = 500 N were, respectively, used in the indentation experiments. The holding time was 15 s. Figure 1b shows the residual imprint left on the surface of specimen (Load-1), the out-of-plane flowing direction (the red arrows), and the interface line.

**Figure 2:**
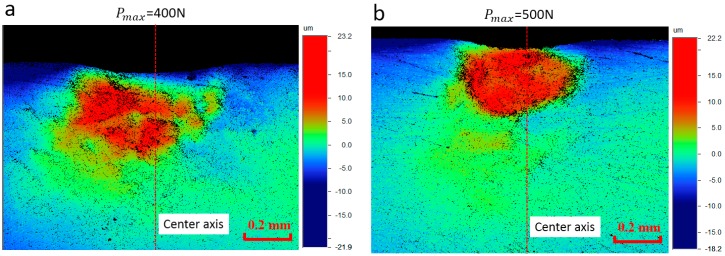
Out-of-plane residual deformation field obtained from indentation experiments using different prescribed loads: (**a**) the prescribed indentation load is 400 N; and (**b**) the prescribed indentation load is 500 N.

The out-of-plane deformation field persisted on the interface surface after unloading is measured using the optical profiling systems (WYKO NT1100, Veeco, Champaign, IL, USA) and the results are, respectively, shown in Figure 2a for Load-1 and Figure 2b for Load-2. This measuring process is based on the white light interferometry theory, and high-resolution 3D surface measurements can be obtained. The orientation of the deformation plane in the experiment can be described by the material coordinate defined in Figure 1. The red dotted line in Figure 2 represents the indentation center axis. It shows that the out-of-plane residual deformation contour deviates from the indentation center axis obviously. We recall that the geometry of the indenter-specimen system is essentially symmetric along the indentation center axis. Therefore, it may be deduced that the asymmetric deformation field persisting on the interface surface is caused by the material plastic anisotropy. This problem will be further investigated in Section 3 and Section 4.

## 3. Methods

### 3.1. Simulation Setup

**Figure 3:**
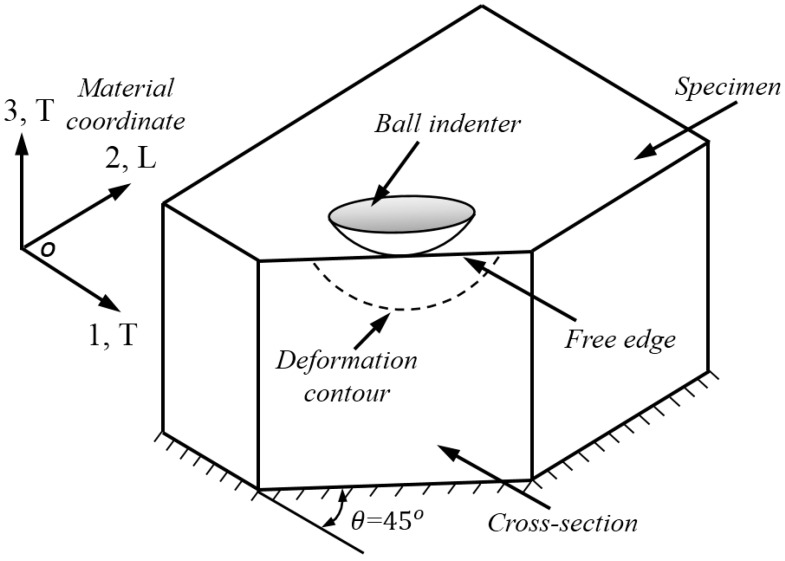
Schematic of the cross-sectional indentation model.

Figure 3 shows the schematic of the cross-sectional indentation model for the anisotropic materials. The anisotropic material considered in the present study exhibits different plastic properties, (e.g., yield stress) along the two orthogonal directions (e.g., longitudinal and transverse), as shown by the material coordinate defined in Figure 3. This material coordinate is used throughout the entire numerical study. The cross-section is designed along the diagonal direction (e.g., 45° to the transverse direction). The indenter is pressed against the free edge of the cross-section, and the loading direction is vertical to the T-L plane. In the indentation process, the cross-section is a free surface with no boundary constraints.

Hill’s plasticity theory [36] is used to describe the deformation behaviors of anisotropic materials in cross-sectional indentation simulation. The general state of this yield function is expressed in Equation (1):

(1) $$f\left(\sigma \right)=\sqrt{F{\left(\sigma_{22}-\sigma_{33}\right)}^{2}+G{\left(\sigma_{33}-\sigma_{11}\right)}^{2}+H{\left(\sigma_{11}-\sigma_{22}\right)}^{2}+2L\tau_{23}^{2}+2M\tau_{31}^{2}+2N\tau_{12}^{2}}$$

where, *F*, *G*, *H*, *L*, *M*, and *N* are anisotropic parameters of the current state of anisotropy [16], and these parameters can be determined by using Equation (2) [38]. The normal and shear yield stress along three orthogonal axes (e.g., 1, 2, and 3 in the material coordinate) are defined as $\sigma_{11}$, $\sigma_{22}$, $\sigma_{33}$ and $\tau_{12}$, $\tau_{31}$,$\;\tau_{23}$, respectively:

(2) $$F=\frac{1}{2}\left(\frac{1}{R_{22}^{2}}+\frac{1}{R_{33}^{2}}-\frac{1}{R_{11}^{2}}\right);G=\frac{1}{2}\left(\frac{1}{R_{33}^{2}}+\frac{1}{R_{11}^{2}}-\frac{1}{R_{22}^{2}}\right);H=\frac{1}{2}\left(\frac{1}{R_{11}^{2}}+\frac{1}{R_{22}^{2}}-\frac{1}{R_{33}^{2}}\right);L=\frac{3}{2R_{23}^{2}};M=\frac{3}{2R_{13}^{2}};N=\frac{3}{2R_{12}^{2}}$$

In Equation (2), the six anisotropic yield stress ratios, $R_{11}$, $R_{22}$, $\;R_{33}$, $\;R_{12}$, $\;R_{13}$, and $R_{23}$ in, respectively, three normal ($R_{11}$, $R_{22}$, and $\;R_{33}$) and three shear ($R_{12}$, $\;R_{13}$, and $R_{23}$) directions are used to quantify the orthogonal anisotropic plasticity, as shown by the material coordinate defined in Figure 3. These six anisotropic constants are inputted by using the POTENTIAL sub-option in ABAQUS software (Dassault, Paris, France) [38]. The R-values are defined by the reference yield stress $\sigma_{Y}$, and the reference shear yield stress $\tau_{Y}$ is defined as $\tau_{Y}=\sigma_{Y}/\sqrt{3}$ according to von Mises criterion. It is noted that the *R*-value defined here is different from the strain ratio *r*, and the latter is usually called the Lankford index [22]. For the anisotropic materials studied here, the other five *R*-values are maintained at unity, and only $R_{22}$ is varied to simulate the in-plane anisotropic plasticity along the 2, L direction. More details about the anisotropic material model studied here can be found in [16,18,38]. The transverse direction is along 1, 3, T axis, and the longitudinal direction is along the 2, L axis. The stress-strain curve along transverse direction is defined as the reference input amount, and the yield stress along the longitudinal axis is determined by using the relation $\sigma_{YL}$ = $R_{22}\sigma_{YT}$. The strain hardening behavior of material is described using the Hollomon hardening law, with a single strain hardening exponent “*n*” for both the longitudinal and transverse directions [18].

### 3.2. Finite Element Modelling

**Figure 4:**
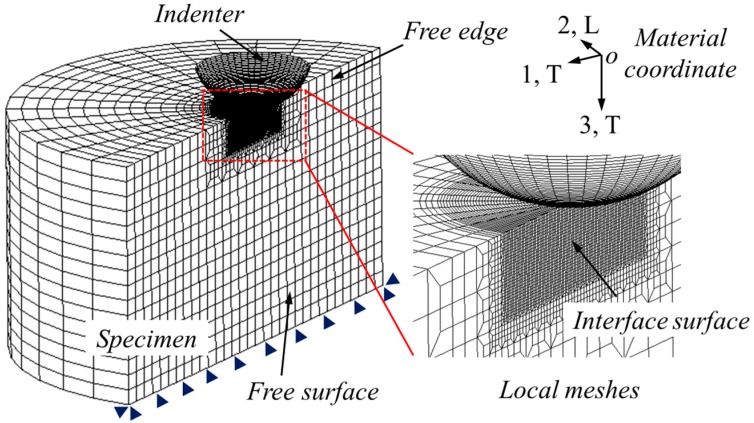
FE model, meshes, and boundary conditions used in the cross-sectional indentation simulation.

The ABAQUS/standard commercial codes [38] were used in cross-sectional indentation simulation. The finite element (FE) model, meshes, and boundary conditions are shown in Figure 4. The spherical indenter was assumed as discrete rigid using the R3D4 element type. Its diameter was 5 mm. The specimen was modeled using the C3D8R element type, and refined meshes were created around the local contact regions between indenter and specimen. Minimum element size in this refined region was 50 µm. Poisson’s ratio of specimen was fixed at 0.3, because Poisson’s ratio is a minor factor in indentation studies [39,40]. The height and radius of specimen was 9.6 mm, and this value is large enough to avoid the influence of outer boundary effects. The total number of elements were 20,232 for the specimen and 4000 for the indenter. Contact friction on the surfaces between the indenter and specimen was defined at 0.1, because the contact friction between metals and diamond is around this value [41,42]. Displacement of the bottom nodes of the specimen was fixed. No boundary constraints were applied on the free surface, so that material during indentation is able to flow freely along the out-of-plane direction (vertical to the free surface, as shown in Figure 3). Vertical displacement of the indenter was controlled by the force increments, up to a prior defined maximum value, and then the withdrawal of the indenter was simulated in one step.

### 3.3. Characterization of the Out-of-Plane Deformation Contour

**Figure 5:**
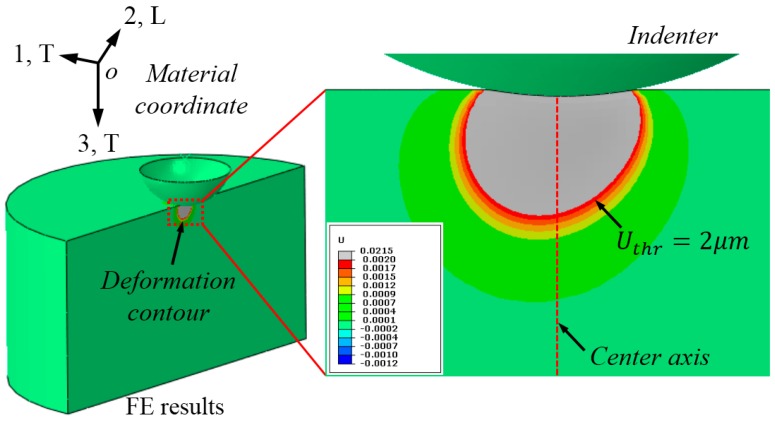
Out-of-plane deformation contour persisting on the cross-section after unloading.

We first performed the FE simulation on a test case, where *E* = 400 GPa, $\sigma_{YT}$ = 200 MPa, *n* = 0.3, and $R_{22}$ = 1.5. The prescribed maximum indentation load is fixed at 800 N. The out-of-plane (vertical to the cross-section) deformation contour of this tested material is shown in Figure 5. The red dotted line is the indentation center axis. Here, a critically-selected displacement threshold value, $U_{thr}$ = 2 µm is used. Figure 5 shows clearly that the bottom shape of the out-of-plane deformation contour is not symmetrical with respect to the indentation center axis, and it deviates toward a specific direction (T-side).

**Figure 6:**
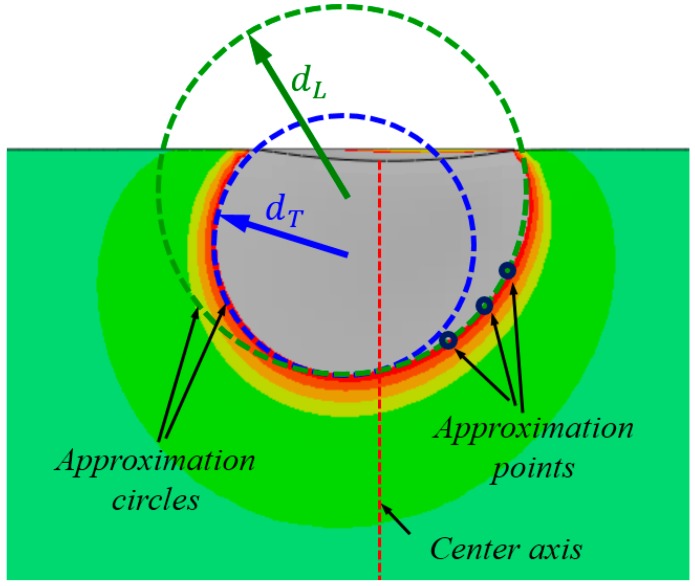
Description of the bottom shape of the out-of-plane deformation contour using two circles.

Figure 6 shows the bottom shape of the out-of-plane deformation contour can be well approximated by using two circles, with diameters $d_{T}$ and $d_{L}$, respectively. This approximation process is very simple. On each side of the deformation contour, select three points on the displacement threshold line. Then, these three points can be used to uniquely identify one approximation circle, as shown in Figure 6. Given the asymmetric shape of the out-of-plane deformation contour persisted on the cross-section after unloading, we hypothesized that the deviation of the bottom shape of the deformation field is caused by the difference of yield stress $\sigma_{YT}$ and $\sigma_{YL}$ in indentation simulation. Additionally, the deformation contour deviates toward an “easier” side. In the study, the relative position of the two approximation circles is described by the ratio of their corresponding diameters $R_{d}$, as $R_{d}$ = $d_{L}/d_{T}$. Therefore, $R_{d}$ can be used to reflect the deviation extent of the bottom shape of the out-of-plane residual deformation contour in cross-sectional indentation.

## 4. Fundamental Relationship between $R_{22}$ and $R_{d}$

### 4.1. Results Obtained from Numerical Simulation

**Figure 7:**
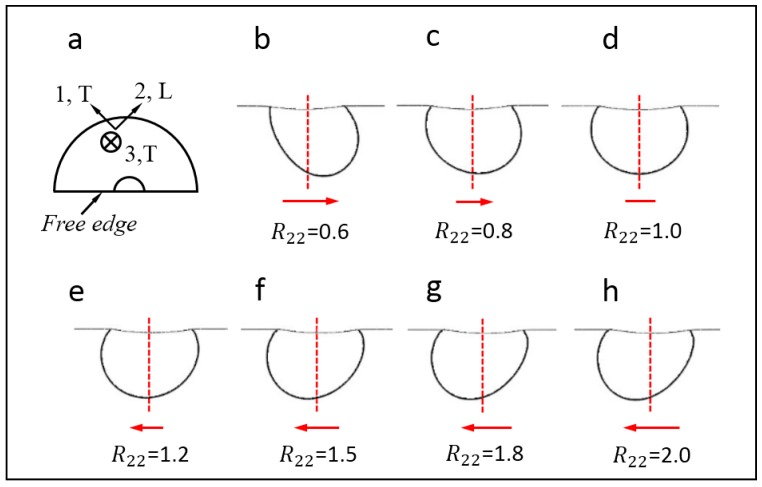
Influence of the $R_{22}$ value on the shape of the out-of-plane deformation contours.

In this section, the possible factor that influences the out-of-plane deformation contour is fully investigated. Figure 7 shows the FE simulation results of a test case, where *E* = 400 GPa, $\sigma_{YT}$ = 200 MPa, *n* = 0.3, and the $R_{22}$ value is varied from 0.6 to 2.0. The prescribed indentation load is 800 N, and the selected displacement threshold value is fixed at $U_{thr}$ = 2 µm. It is clearly shown in Figure 7 that the deviation direction (see the red arrows) of the out-of-plane deformation contour changes monotonously with $R_{22}$ increases. When $R_{22}<\;$1, the contour line deviates toward the L side, while its deviation direction changes gradually to the T side when $R_{22}>\;$1. Additionally, when $R_{22}$ = 1, the contour line is symmetric. Therefore, the deformation contour always deviates toward an “easier” side, where the yield stress is lower.

**Figure 8:**
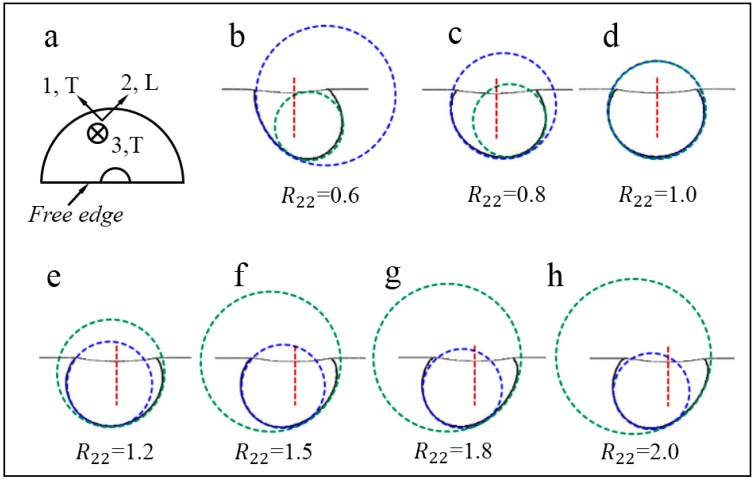
Evolution of the relative position between two approximation circles as $R_{22}$ increases.

Figure 8 shows the evolution of the relative position of two approximation circles with $R_{22}$ increases. It shows clearly that when $R_{22}<\;1$, $d_{T}$ is larger than $d_{L}$ ($R_{d}<1$) while $d_{T}$ becomes smaller than $d_{L}$ ($R_{d}>1$) when $R_{22}>1$. Additionally, when the material is isotropic ($R_{22}=1$), the two approximation circles are nearly coincident ($d_{T}$ = $d_{L}$), and $R_{d}=1$. Therefore, there exists a strong relevance between the bottom shape of the out-of-plane deformation contour ($R_{d}$ value) and the plastic anisotropy parameter $R_{22}$.

**Figure 9:**
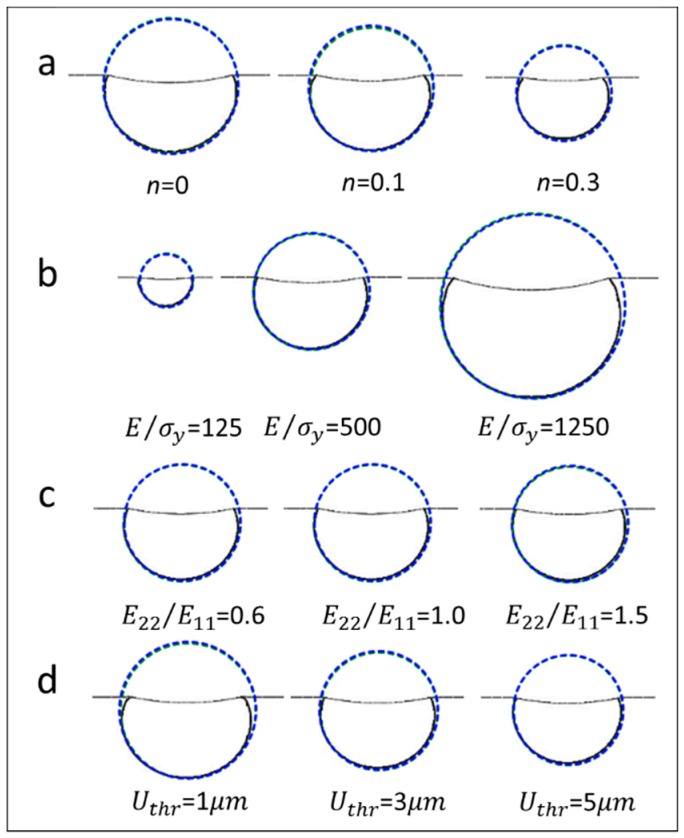
Influence of the strain hardening exponent, yield strain, elastic anisotropy, and the selected displacement threshold value on the shape of contour lines (for all the simulations in this figure, $R_{22}$ is fixed at 1.0).

Figure 9 shows the FE simulation results of another test case, where the influence of the strain hardening exponent, yield strain, elastic anisotropy, and the selected displacement threshold value, on the shape of out-of-plane deformation contour is systematically investigated. In all the simulations, the material is assumed as isotropic ($R_{22}$ = 1.0). In Figure 9, it shows all the deformation contours are symmetric, as it was depicted in Figure 7d. Although, the magnitudes of these contour lines are different, the two approximation circles are nearly coincident.

**Figure 10:**
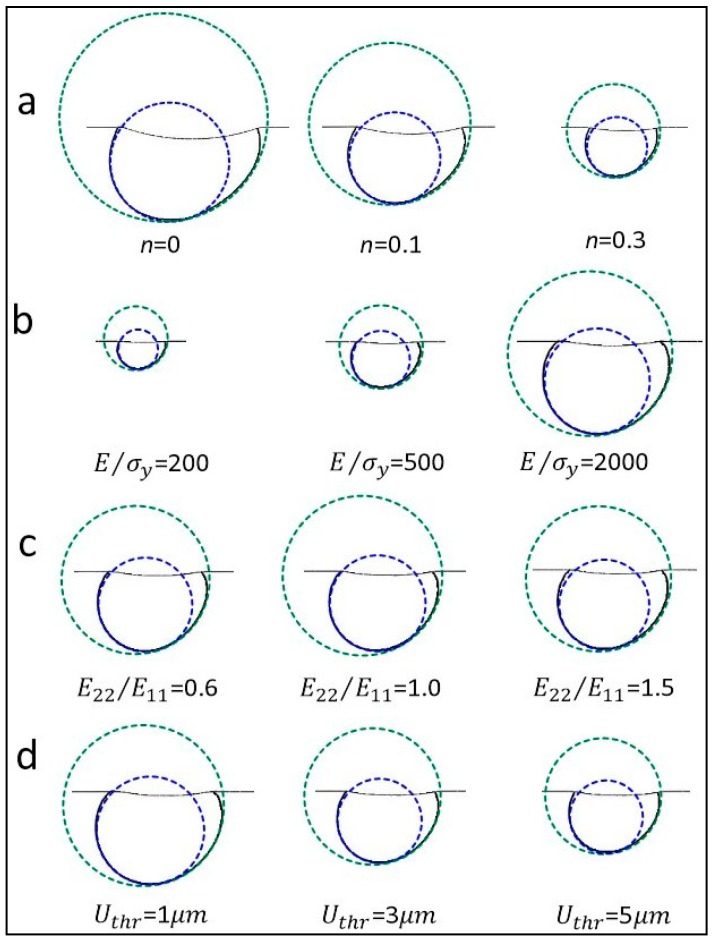
Influence of the strain hardening exponent, yield strain, elastic anisotropy, and the selected displacement threshold value on the relative position between two approximation circles (for all the simulations in this figure, $R_{22}$ is fixed at 1.5).

Similar result can be found in Figure 10, where the $R_{22}$ value in all these simulations is fixed at 1.5. Figure 10 shows that the magnitudes of these contour lines are different, while the relative position of the two approximation circles are nearly the same, as it was depicted in Figure 8f. Therefore, the results indicate the strain hardening exponent, yield strain, elastic anisotropy, and the selected displacement threshold value hardly influence the bottom shape of the out-of-plane deformation contour. It is noted that the orientation of the deformation planes shown in Figure 7, Figure 8, Figure 9 and Figure 10 are the same.

**Figure 11:**
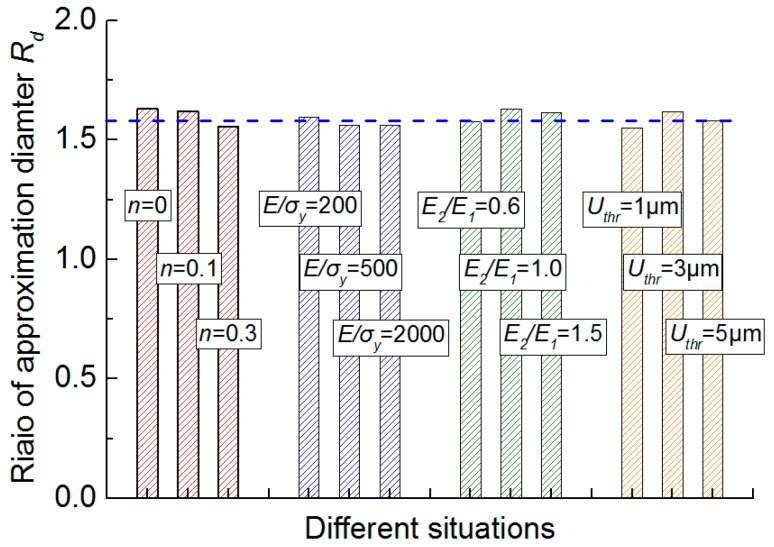
Influence of the strain hardening exponent, yield strain, elastic anisotropy, and the selected displacement threshold value on the diameter ratio, $R_{d}$ of the two approximation circles.

Figure 11 shows the extracted $R_{d}$ values using all the contour lines in Figure 10a–d, respectively, under four different situations. Figure 11 shows the $R_{d}$ values are very close. The small difference of $R_{d}$ values in Figure 11 is probably caused by some uncertain factors, e.g., numerical oscillations in the FE simulation and the approximation error. Therefore, the result indicates the bottom shape of the out-of-plane deformation contour is only dependent on the $R_{22}$ value, and independent of the strain hardening exponent, yield strain, elastic anisotropy and the selected displacement threshold value.

**Figure 12:**
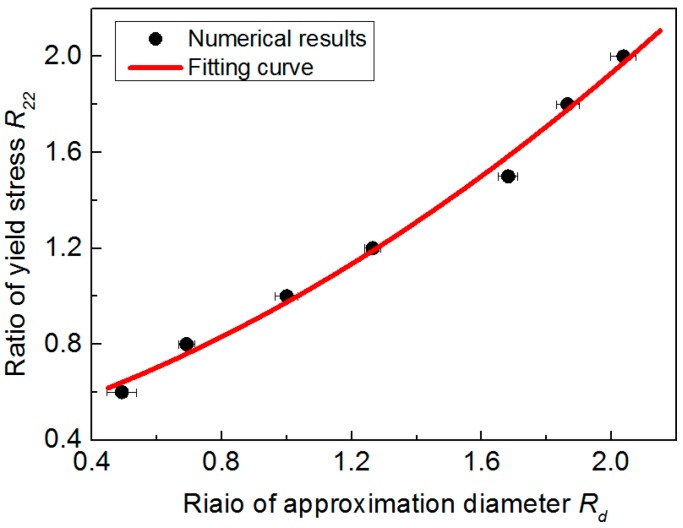
Relationship between $R_{22}$ and $R_{d}$.

Figure 12 shows the relationship between $R_{22}$ and $R_{d}$. The result is extracted from Figure 8, and its relationship is approximated by using a second polynomial function, as expressed in Equation (3). Its shows the relationship between $R_{22}$ and $R_{d}$ is monotonic. Therefore, Equation (3) can be used to uniquely estimate the anisotropic parameter $R_{22}$, when the $R_{d}$ value is obtained from the cross-sectional indentation.

(3) $$R_{22}=\;0.1972R_{d}^{2}+0.3639R_{d}+0.4137$$

### 4.2. Comparison with the Experiment

**Figure 13:**
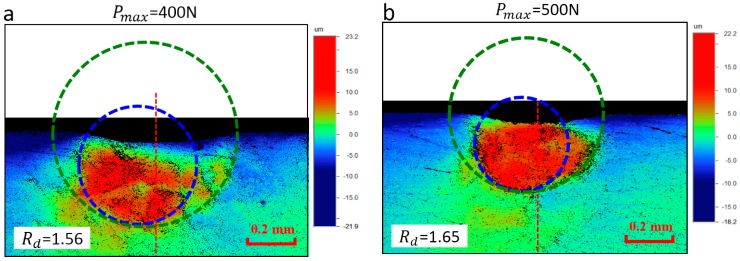
Out-of-plane deformation contour: (**a**) The prescribed indentation load is 400 N; (**b**) The prescribed indentation load is 500 N.

**Figure 14:**
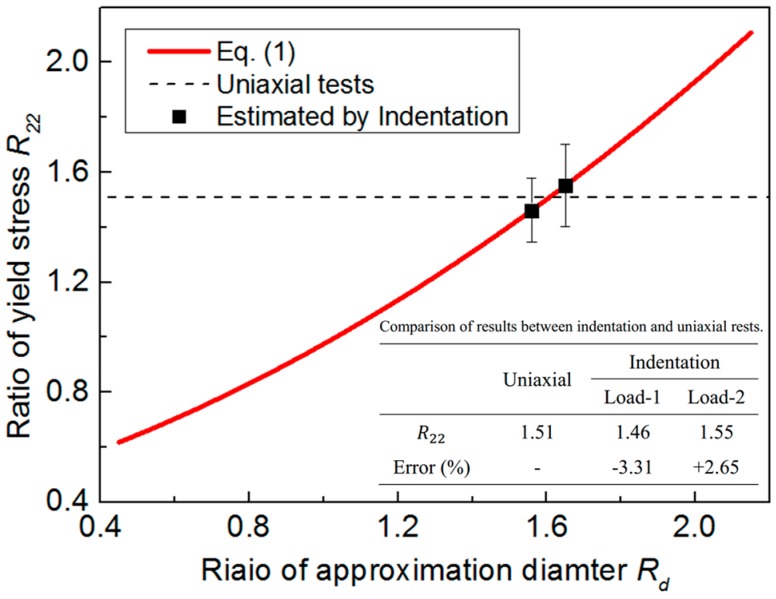
Comparison of the estimated $R_{22}$ value between indentation and uniaxial tests.

Figure 13 shows the approximation results of the out-of-plane residual deformation field using two circles. Here, the selected $U_{thr}$ value is about 10 µm. Results show the $R_{d}$ values are about 1.56 for Load-1, and 1.65 for Load-2. Figure 14 shows the estimated $R_{22}$ value using Equation (3), and its comparison with the uniaxial test data. A good agreement can be found, as shown in Figure 14. The error of the $R_{22}$ value between indentation and uniaxial tests are about −3.31% under Load-1, and +2.65% under Load-2. Results indicate the proposed method in the present study is very effective, and the reproducibility is good. Additionally, an indentation load of 500 N provides a more accurate result. In the proposed methodology it is very important to obtain the effective experimental data, e.g., the residual deformation field since the fitting of the circle is dependent on the selected three approximation points. This approximation is reliable when the displacement contour is well-defined. In the numerical simulation, the displacement contours are obtained from the FE simulation and they are well-defined while, in the experiment, the fitting result may be influenced by the quality of the experimental data, because the displacement contour can be influenced by some uncertainty factors, such as the prescribed indentation load, surface evenness and roughness, the glue thickness, and so on. Thus, it is essential to improve the quality of the experimental data.

In the experiment, the indentation load should be properly selected. On the one hand, elastic plastic transition [5] will occur with load increases. Thus, the load should be larger, so that the deformation is plasticity dominates, while too higher a load may result in the occurrence of some uncertain factors, such as the fracture along the edge. Therefore, it is suggested here that the load can be optimized in the real experiment using the trial and error method to obtain a better result, e.g., a well-defined displacement contour. On the other hand, it should be noted that the quality of the experimental data depends on the combined effects between the load and the other uncertain experiment factors, e.g., surface roughness and glue thickness. In the experiments, two indentation loads, 400 N and 500 N, were selected, because they gave good experimental data. When the experiment error (e.g., surface evenness and roughness) is fixed at a certain level, a higher indentation load may induce a larger out-of-plane displacement value, thus it can reduce the influence of these uncertain factors on the quality of the displacement contour. However, this effect may be simultaneously influenced by the glue thickness when the displacement value is too large. From this aspect, the current experiment condition has a lot of room for the improvement, e.g., higher experiment precision, or a more properly designed glue thickness value. These are open questions, and will be further studied in our future work.

## 5. Discussion

In the present study, a novel approach is established to estimate the plastic anisotropy of bulk metallic materials by examining the bottom shape of the out-of-plane residual deformation contour in cross-sectional indentation. It is noted that the indentation size in the present study is within the macro-scale (e.g., the indenter radius is 2.5 mm), so the indentation size-effect is not considered. Additionally, the top and bottom surfaces of the specimen should be flat in order to avoid the tilt of the specimen during the indentation loading process. The influence of tilt angle, indenter offset, glue thickness, and the other possible factors, e.g., size-effect, grain size, on the shape of the out-of-plane deformation field will be further investigated in our future work. In numerical computations, a relatively simple yield criterion, e.g., Hill’s plasticity theory [36], with isotropic strain hardening behaviors was used. Although more complex yield criterion may bringing more accurate numerical results, e.g., Barlat 91 criterion [43], this problem is out of the current research scope. In addition, the influence of element size on the simulation output was systematically investigated. Results indicate the more refined meshes in the simulation model are able to give a more precise description of the residual deformation field, while this improvement is very limited, and the computation burden will increase accordingly. Additionally, the selected element size in the present study is sufficient to give a very precise description of the material deformation field.

The established methodology in the present study can be used to estimate the plastic anisotropy of specimens with finite volumes, where the uniaxial tests are not applicable. Although the cross-sectional indentation technique has been used to characterize the mechanical behaviors of a large number of materials, e.g., fracture toughness of thermal barrier coatings [8] and the shear band of metallic glasses [44], its application on the characterization of mechanical behaviors of in-plane anisotropic materials is rare. Perhaps, the experiment process of this method may be as laborious as the conventional uniaxial tests. However, it does provide a novel method to uniquely correlate the plastic anisotropy parameter with the measurable material response, e.g., the out-of-plane deformation field in cross-sectional indentation. For the research purposes, it can be used to probe the relationship between anisotropy, microtexture, microstructure, and processing behaviors of materials [17,31,32,33], particularly in the situations where the information of the entire indentation loading history (e.g., the load-displacement curve data) is not available.

## 6. Conclusions

We presented in this paper a methodology for obtaining the plastic anisotropy of metal materials using cross-sectional indentation. The study focuses on the bulk metallic materials which possess different yield stress along the longitudinal and transverse directions. A computational model was built to simulate the spherical indentation on the free edge of the cross-section. Results obtained from numerical simulation revealed that the bottom shape of the out-of-plane deformation field persisted on the cross-section after unloading is only sensitive to the extent of material plastic anisotropy, and insensitive to strain hardening exponent, yield strain, elastic anisotropy and the selected displacement threshold value. Based on the parametric FE study, an explicit equation is established to determine the material plastic anisotropy using the characteristic parameter of the out-of-plane residual deformation contour in the cross-sectional indentation. The effectiveness of this method is verified by application on magnesium alloy AZ31B, and the anisotropic parameter estimated from indentation and uniaxial tests show good agreement.

## Figures and Tables

**Table 1:** Chemical composition of magnesium alloy AZ31B.

Chemistry	Al	Zn	Mn	Si	Fe	Cu	Ni	Mg
wt %	2.5–3.5	0.6–1.4	0.2–1.0	≤0.80	≤0.003	≤0.01	≤0.001	Balance

**Table 2:** Uniaxial compression experimental data of magnesium alloy AZ31B.

AZ31B	*E* (GPa)	$\sigma_{y}$ (MPa)	*n*
1, T	33.5	64.2	0.287
3, T	34.5	68.5	0.260
2, L	32.3	100.5	0.268

## References

[1] Tabor D. (1951). The Hardness of Metals.

[2] Wang W., Wu J., Hui Y., Kun Z., Zhan X., Guo R. (2016). Identification of elastic-plastic properties of metal materials by using the residual imprint of spherical indentation. Mater. Sci. Eng. A.

[3] Zambaldi C., Raabe D. (2010). Plastic anisotropy of γ-TiAl revealed by axisymmetric indentation. Acta Mater..

[4] Oliver W.C., Pharr G.M. (1992). An improved technique for determining hardness and elastic-modulus using load and displacement sensing indentation experiments. J. Mater. Res..

[5] Patel D.K., Kalidindi S.R. (2016). Correlation of spherical nanoindentation stress-strain curves to simple compression stress-strain curves for elastic-plastic isotropic materials. Acta Mater..

[6] Moussa C., Hernot X., Bartier O., Delattre G., Collin J.M., Mauvoisin G. (2016). Mechanical characterization of carbonitrided steel with spherical indentation using the average representative strain. Mater. Des..

[7] Roy T.K. (2015). Assessing hardness and fracture toughness in sintered zinc oxide ceramics through indentation technique. Mater. Sci. Eng. A.

[8] Wang X., Wang C.J., Atkinson A. (2010). Interface fracture toughness in thermal barrier coatings by cross-sectional indentation. Acta Mater..

[9] Attar H., Ehtemam-Haghighi S., Kent D., Okulov I.V., Wendrock H., Bnisch M., Volegov A.S., Calin M., Eckert J., Dargusch M.S. (2017). Nanoindentation and wear properties of Ti and Ti-TiB composite materials produced by selective laser melting. Mater. Sci. Eng. A.

[10] Ehtemam-Haghighi S., Cao G., Zhang L.C. (2017). Nanoindentation study of mechanical properties of Ti based alloys with Fe and Ta additions. J. Alloys Comp..

[11] Ehtemam-Haghighi S., Prashanth K.G., Attar H., Chaubey A.K., Cao G.H., Zhang L.C. (2016). Evaluation of mechanical and wear properties of Ti-xNb-7Fe alloys designed for biomedical applications. Mater. Des..

[12] Vlassak J.J., Nix W.D. (1994). Measuring the elastic properties of anisotropic materials by means of indentation experiments. J. Mech. Phys. Solids.

[13] Bocciarelli M., Bolzon G., Maier G. (2005). Parameter identification in anisotropic elastoplasticity by indentation and imprint mapping. Mech. Mater..

[14] Nakamura T., Gu Y. (2007). Identification of elastic-plastic anisotropic parameters using instrumented indentation and inverse analysis. Mech. Mater..

[15] Bolzon G., Talassi M. (2013). An effective inverse analysis tool for parameter identification of anisotropic materials. Int. J. Mech. Sci..

[16] Yonezu A., Yoneda K., Hirakata H., Sakihara M., Minoshima K. (2010). A simple method to evaluate anisotropic plastic properties based on dimensionless function of single spherical indentation-Application to SiC whisker-reinforced aluminum alloy. Mater. Sci. Eng. A.

[17] Kalkhoran S.M., Choi W.B., Gouldstone A. (2012). Estimation of plastic anisotropy in Ni–5% Al coatings via spherical indentation. Acta Mater..

[18] Wang M., Wu J., Zhan X., Guo R., Hui Y., Fan H. (2016). On the determination of the anisotropic plasticity of metal materials by using instrumented indentation. Mater. Des..

[19] Garmestani H., Kalidindi S.R., Williams L., Bacaltchuk G.M., Fountain C., Lee E.W., Es-Said O.S. (2002). Modeling the evolution of anisotropy in Al-Li alloys: Application to Al-Li 2090-T8E41. Int. J. Plast..

[20] Yoshida F., Hamasaki H., Uemori T. (2013). A user-friendly 3D yield function to describe anisotropy of steel sheets. Int. J. Plast..

[21] Zhang H., Diehl M., Roters F., Raabe D. (2015). A virtual laboratory using high resolution crystal plasticity simulations to determine the initial yield surface for sheet metal forming operations. Int. J. Plast..

[22] Lankford W.T., Snyder S.C., Bauscher J.A. (1950). New criteria for predicting the press performance of deep drawing sheets. Trans. Am. Soc. Met..

[23] Wang Y.N., Huang J.C. (2007). The role of twinning and untwining in yielding behavior in hot-extruded Mg-Al-Zn alloy. Acta Mater..

[24] Yan H., Chen R.S., Han E.H. (2010). Room-temperature ductility and anisotropy of two rolled Mg–Zn–Gd alloys. Mater. Sci. Eng. A.

[25] Yi S., Bohlen J., Heinemann F., Letzig D. (2010). Mechanical anisotropy and deep drawing behavior of AZ31 and ZE10 magnesium alloy sheets. Acta Mater..

[26] Tang W., Huang S., Li D., Peng Y. (2015). Mechanical anisotropy and deep drawing behaviors of AZ31 magnesium alloy sheets produced by unidirectional and cross rolling. J. Mater. Process. Technol..

[27] Kim W.J., Yoo S.J., Chen Z.H., Jeong H.T. (2009). Grain size and texture control of Mg–3Al–1Zn alloy sheet using a combination of equal-channel angular rolling and high-speed-ratio differential speed-rolling processes. Scr. Mater..

[28] Li X., Al-Samman T., Gottstein G. (2011). Mechanical properties and anisotropy of ME20 magnesium sheet produced by unidirectional and cross rolling. Mater. Des..

[29] Stanford N., Barnett M.R. (2008). The origin of “rare earth” texture development in extruded Mg-based alloys and its effect on tensile ductility. Mater. Sci. Eng. A.

[30] Stanford N., Atwell D., Beer A., Davies C., Barnett M.R. (2008). Effect of microalloying with rare-earth elements on the texture of extruded magnesium-based alloys. Scr. Mater..

[31] Crooks R., Wang Z., Levit V.I., Shenoy R.N. (1998). Microtexture, microstructure and plastic anisotropy of AA2195. Mater. Sci. Eng. A.

[32] Alexander B.-B., Carl B., Franck A.T.G., Daniel L. (2016). Modelling of anisotropy for Al–Li 2099 T83 extrusions and effect of precipitate density. Mater. Sci. Eng. A.

[33] Alexander B.-B., Carl B., Franck A.T.G., Daniel L., Julien B., Mathieu B. (2014). Characterization of Al-Li 2099 extrusions and the influence of fiber texture on the anisotropy of static mechanical properties. Mater. Sci. Eng. A.

[34] Rioja R.J. (1998). Fabrication method to manufacture isotropic Al-Li alloys and products for space and aerospace applications. Mater. Sci. Eng. A.

[35] Yonezu A., Kuwahara Y., Yoneda K., Hirakata H., Minoshima K. (2009). Estimation of the anisotropic plastic property using single spherical indentation-An FEM study. Comp. Mater. Sci..

[36] Hill R. (1948). A theory of the yielding and plastic flow of anisotropic metals. Proc. R. Soc. A.

[37] Donohue B.R., Ambrus A., Kalidindi S.R. (2012). Critical evaluation of the indentation data analyses methods for the extraction of isotropic uniaxial mechanical properties using finite element models. Acta Mater..

[38] (2009). ABAQUS.

[39] Zhao M., Ogasawara N., Chiba N., Chen X. (2006). A new approach to measure the elastic-plastic properties of bulk materials using spherical indentation. Acta Mater..

[40] Hernot X., Moussa C., Bartier O. (2014). Study of the concept of representative strain and constraint factor introduced by Vickers indentation. Mech. Mater..

[41] Bowden F.P., Tabor D. (2001). The Friction and Lubrication of Solids.

[42] Bucaille J.L., Stauss S., Felder E., Michler J. (2003). Determination of plastic properties of metals by instrumented indentation using different sharp indenters. Acta Mater..

[43] Barlat F., Lege D.J., Brem J.C. (1991). A six-component yield function for anisotropic materials. Int. J. Plast..

[44] Zhang H., Jing X., Subhash G., Kecskes L.J., Dowding R.J. (2005). Investigation of shear band evolution in amorphous alloys beneath a Vickers indentation. Acta Mater..

